# An Adaptive Mobile Health System to Support Self-Management for Persons With Chronic Conditions and Disabilities: Usability and Feasibility Studies

**DOI:** 10.2196/12982

**Published:** 2019-04-25

**Authors:** I Made Agus Setiawan, Leming Zhou, Zakiy Alfikri, Andi Saptono, Andrea D Fairman, Brad Edward Dicianno, Bambang Parmanto

**Affiliations:** 1 Department of Health Information Management School of Health and Rehabilitation Sciences University of Pittsburgh Pittsburgh, PA United States; 2 Department of Computer Science Udayana University Badung, Bali Indonesia; 3 Department of Occupational Therapy School of Health and Rehabilitation Sciences MGH Institute of Health Professions Boston, MA United States; 4 Department of Rehabilitation Science and Technology School of Health and Rehabilitation Sciences University of Pittsburgh Pittsburgh, PA United States; 5 Department of Physical Medicine and Rehabilitation School of Medicine University of Pittsburgh Pittsburgh, PA United States

**Keywords:** mHealth, adaptive mHealth, mobile apps, telemedicine, personalized medicine, self-management, self-care, caregivers, chronic disease, persons with disabilities

## Abstract

**Background:**

Persons with chronic conditions and disabilities (PwCCDs) are vulnerable to secondary complications. Many of these secondary complications are preventable with proactive self-management and proper support. To enhance PwCCDs' self-management skills and conveniently receive desired support, we have developed a mobile health (mHealth) system called iMHere. In 2 previous clinical trials, iMHere was successfully used to improve health outcomes of adult participants with spina bifida and spinal cord injury. To further expand use of iMHere among people with various types of disabilities and chronic diseases, the system needs to be more adaptive to address 3 unique challenges: 1) PwCCDs have very diverse needs with regards to self-management support, 2) PwCCDs’ self-management needs may change over time, and 3) it is a challenge to keep PwCCDs engaged and interested in long-term self-management.

**Objective:**

The aim of this study was to develop an *adaptive* mHealth system capable of supporting long-term self-management and adapting to the various needs and conditions of PwCCDs.

**Methods:**

A scalable and adaptive architecture was designed and implemented for the new version, iMHere 2.0. In this *scalable* architecture, a set of mobile app modules was created to provide various types of self-management support to PwCCDs with the ability to add more as needed. The *adaptive* architecture empowers PwCCDs with personally relevant app modules and allows clinicians to adapt these modules in response to PwCCDs’ evolving needs and conditions over time. Persuasive technologies, social support, and personalization features were integrated into iMHere 2.0 to engage and motivate PwCCDs and support long-term usage. Two initial studies were performed to evaluate the usability and feasibility of the iMHere 2.0 system.

**Results:**

The iMHere 2.0 system consists of cross-platform client and caregiver apps, a Web-based clinician portal, and a secure 2-way communication protocol for providing interactions among these 3 front-end components, all supported by a back-end server. The client and caregiver apps have 12 adaptive app modules to support various types of self-management tasks. The adaptive architecture makes it possible for PwCCDs to receive personalized app modules relevant to their conditions with or without support from various types of caregivers. The personalization and persuasive technologies in the architecture can be used to engage PwCCDs for long-term usage of the iMHere 2.0 system. Participants of the usability study were satisfied with the iMHere 2.0 client app. The feasibility evaluation revealed several practical issues to consider when implementing the system on a large scale.

**Conclusions:**

We developed an adaptive mHealth system as a novel method to support diverse needs in self-management for PwCCDs that can dynamically change over time. The usability of the client app is high, and it was feasible for PwCCDs to use in supporting personalized and evolving self-care needs.

## Introduction

### Background

Chronic conditions have become one of the greatest challenges for the US health care system [1]. According to a report published in 2017, 60% of US adults had at least 1 chronic condition, 42% had more than 1 chronic condition, and 12% had 5 or more chronic conditions [1]. People with chronic conditions account for 90% of health care spending [1,2], and the spending on health care services increases with the number of chronic conditions [1]. More specifically, the 12% of Americans with 5 or more chronic conditions account for 41% of total health care spending. In addition, about a quarter of persons with chronic conditions have some type of disability (hereafter, *persons with chronic conditions and disabilities* —PwCCDs, in this paper). These disabilities limit daily activities and social participation, can significantly impact quality of life, and can increase susceptibility to developing secondary complications [1,2].

One population of PwCCDs is individuals with pediatric onset or congenital malformations of the brain and spinal cord, including individuals with spina bifida (SB) and cerebral palsy (CP). SB is the most common permanently disabling birth defect in the United States [3]. SB refers to the incomplete development of the neural tube closure, which results in loss of sensation and major muscle weakness in the lower portion of the body [4]. Individuals with SB are at substantial risk for paralysis, bladder dysfunction and gastrointestinal issues, and orthopedic abnormalities [4]. CP involves impairment of motor function as a result of brain damage, which often occurs to the cerebral motor cortex owing to nonprogressive disturbances during brain development in fetuses or infants. Individuals with CP experience varying levels of activity limitation [5,6]. Other issues include gastrointestinal and urinary problems, abnormal neurologic control, abnormal sensation and perception, mental health issues, epilepsy, and intellectual disability [5-8]. Some of these conditions can lead to other secondary conditions, such as urinary tract infections and pressure ulcers [9-11]. These secondary complications can have a major impact on all aspects of an individual’s life.

Self-management support has been found to be useful for PwCCDs to prevent secondary complications [12-14]. However, it is challenging to provide desired self-management support to PwCCDs for several reasons.

First, many different chronic conditions exist and these vary in severity and differ broadly in terms of characteristics. Consequently, PwCCDs have *very diverse needs with regard to self-management support*.

Second, the phases of chronic conditions and life circumstances change over time [15]. Many chronic conditions become worse over time without treatment or become better with proper treatment. PwCCDs also commonly experience change in their emotional states, such as frustration and depression [16]. When these changes happen, adjustments in the self-management strategy need to take place. For instance, an individual may need certain self-management support to prevent pressure ulcers, but once an ulcer develops, a different self-management routine may be needed to treat it. Moreover, different individuals may need to follow different treatments for pressure ulcers depending on their own particular circumstances, for example, the location or depth of the wound may necessitate different treatment strategies. In other words, *PwCCDs’ self-management needs may change over time*. Therefore, the strategies to support self-management vary by individual and within the individual over the course of a lifetime.

Third, the majority of chronic conditions and disabilities are inherently long-lasting, generally lifelong [17]. Therefore, the self-management will be long-term as well [16]. As a result, it is a challenge *to keep PwCCDs engaged and interested in long-term self-management*.

A mobile health (mHealth) intervention is one approach to encourage proactive self-management skills and improve well-being to reduce the development of secondary complications and health care costs [18-20]. Several studies have evaluated the benefit of mHealth in managing chronic conditions [21-24], and the results indicated that mHealth could provide better adherence to intervention regimens, such as compliance with taking medications, and better self-tracking capability to support self-management. Therefore, mHealth showed promise in promoting health-related activities.

Previous studies have also indicated that social influence from caregivers would be helpful for long-term adherence to self-management [25,26]. The integration of persuasive technologies to induce action or foster belief through encouragement or inspiration may help to maintain the level of engagement in a long-term care plan [27-29]. Moreover, a recent study revealed the importance of personalization and adaptability of digital support in different contexts for supporting self-management of PwCCDs [30].

However, currently there is no integrated mHealth system available for adaptive and long-term self-management support, especially for PwCCDs.

### Objectives

The aim of this study was to develop an adaptive mHealth system, Interactive Mobile Health and Rehabilitation (iMHere) 2.0, which can support self-management for long-term usage and allow PwCCDs to receive personalized and adaptive treatment strategies according to their needs and conditions. It is expected that this system will provide the ability to personalize the treatment strategies at any time according to the PwCCDs’ specific situation, which may eventually prevent many secondary conditions and improve quality of life.

## Methods

### Overview

**Figure 1:**
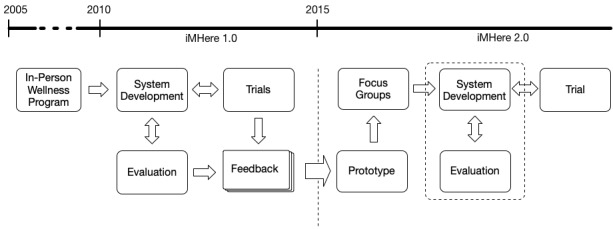
Timeline and workflow of the iMHere 2.0 system development.

This project utilized the information and experience obtained from previous studies to create an adaptive mHealth system [31-39]. The system incorporates persuasive technologies and social support to maintain the level of engagement of PwCCDs [25-29]. As we desired to expand use of the system to different diagnoses and demographics, a user-centered approach was used to gather knowledge of additional requirements [40]. The iMHere 2.0 system was iteratively designed and incrementally developed and evaluated with users involved in all stages. Figure 1 shows the general workflow of the iMHere 2.0 system development. PwCCDs with different diagnoses and demographics were involved in all of these steps, providing requirements, feedback, and comments.

The findings from the previous studies and the themes elicited from the focus groups are highly consistent [31-40]. These findings and themes revealed that a significant architecture change was necessary to improve the iMHere system. Following are several major changes incorporated in the iMHere 2.0 system in response:

- Redesign of the overall architecture of the system to make it *scalable* and convenient to add more components and new mobile app modules into the system.
- Implementation of *adaptive intervention* approaches to allow the system to address different characteristics and needs in different individuals and within individuals over time [41].
- Incorporation of social support from caregivers via a mobile app to maintain PwCCDs’ long-term engagement in the mHealth system [40].
- Ability of the mHealth apps to run on different platforms, including Android, iOS, and Windows Phone systems, which makes the iMHere 2.0 system available to almost any PwCCDs [42].
- Enhancement of the existing mobile app modules (iMHere 1.0 with 5 modules) and addition of 7 new modules to meet the need for diverse types of self-management support.
- Addition of accessibility features to increase the ease of use of the mobile app, especially for individuals with fine motor and visual impairments [36].

### A Scalable System Architecture

**Figure 2:**
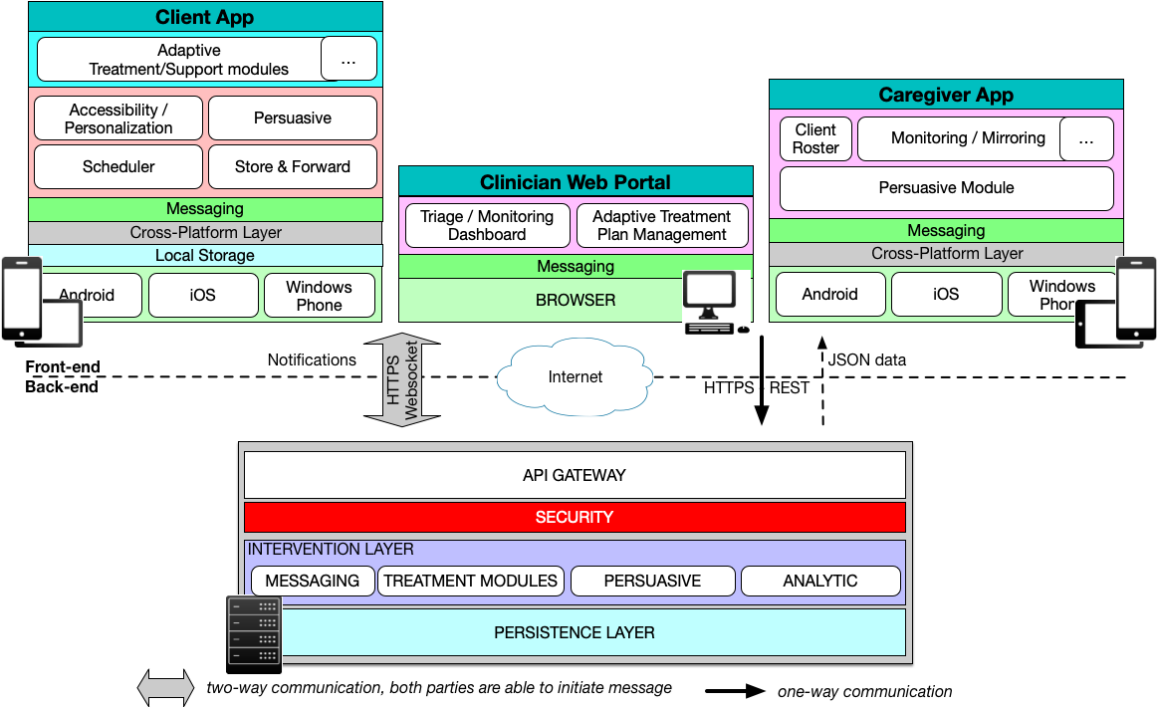
The architecture of the iMHere 2.0 system.

The iMHere 2.0 system consists of 5 components: a client app, a caregiver app, a Web-based clinician portal, a back-end server, and a 2-way secure communication protocol (see Figure 2). The first 3 are user-facing front-end components.

The client app is used by the PwCCDs (client) for self-management via a set of modules tailored to the PwCCDs’ needs. The client app can synchronize PwCCDs’ care data among multiple personal mobile devices, the caregiver’s app, and the Web-based portal.

The caregiver app is for caregivers (family members, friends, and professional caregivers) to provide monitoring and social support to the PwCCDs. Both the client and caregiver apps can run on multiple mobile platforms including Android, iOS, and Windows Phone systems.

The Web-based portal is for clinicians to monitor the PwCCDs’ progress and prepare the intervention regimens. All data from the client app are presented in the Web-based portal to help clinicians evaluate the PwCCDs’ progress and then perform adjustment on the intervention regimens if needed. In other words, according to the PwCCDs’ current situation, the clinician can easily update their intervention strategies for an individual PwCCD via the Web-based portal and synchronize it to the client and caregiver apps in real time. The 2-way secure communication protocol enables all 3 front-end components to communicate in real time.

All 3 components are connected and supported by the back-end server, which has been designed to be highly scalable using microservices. In microservices, applications are composed of multiple small independent services that can be implemented and deployed independently [43]. This approach offered us the possibility of extending the iMHere 2.0 system without disturbing the other existing components. For example, one can add a new app module into the iMHere 2.0 system for PwCCDs with needs that are unavailable in the current version; one can also link the iMHere 2.0 system with an external electronic health record system to exchange patient information.

This scalable system architecture provides the foundation for creating various types of self-management app modules to meet the highly diverse needs of PwCCDs. In the next section, we have described the 12 app modules created on this scalable architecture to demonstrate this feature.

### A Web-Based Clinician Portal for Personalized and Adaptive Interventions

**Figure 3:**
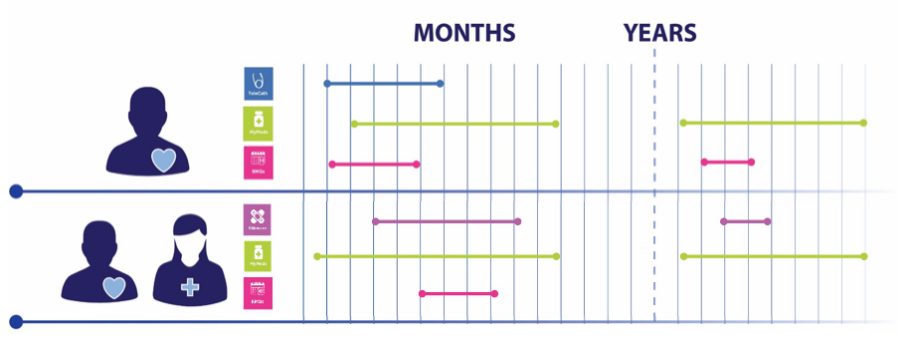
Different support between clients and for one client across time. The icon and color of the line correspond to a specific app module and the length of the line shows the duration of using each app module.

The primary purpose of the Web-based portal is to allow clinicians to prescribe personalized treatment plans, monitor the PwCCDs’ conditions and their adherence to the intervention regimens, perform adjustments on the regimens according to the progress of the PwCCDs (Figure 3), and communicate with the PwCCDs via instant messaging. All information regarding PwCCDs’ adherence to intervention regimens and data from the client app are presented in the portal to help clinicians evaluate PwCCDs’ progress. Communication with PwCCDs can be engaged in through the messaging feature, if there is any issue that clinicians want to discuss with PwCCDs. Once a clinician adjusts a PwCCD’s treatment plan on the Web-based portal, the changes are immediately pushed to the client and caregiver apps.

### Client App Modules for Diverse Self-Management Needs

The current version of the iMHere 2.0 client app has 12 app modules. They were created according to feedback from clinicians, caregivers, and PwCCDs themselves [31-40]. In total, 5 of these app modules (MyMeds, BMQ, TeleCath, Mood, and Skincare) are an updated version of the 5 existing modules in the iMHere 1.0 system.

The major update on these 5 app modules included providing more flexible ways for the user to arrange schedules and improving the accessibility. The updated app modules provide the capability to create a fine-grain schedule for reminders, including the ability to make different schedules for weekdays and the weekend as well as hour-based schedules. A customized camera feature was added in the Skincare module to guide the user in taking more consistent wound pictures, and a binding physical button was added to trigger the camera in addition to the soft-button on the screen [34].

These 5 updated app modules are able to provide support for medication management, bowel management, bladder self-catheterization, mood assessment, and skin problem reporting and tracking. For instance, the new version of the MyMeds module provides a mechanism for tracking medication that is taken on an as needed basis, such as medication for pain.

As the client app will be used for long-term care, the screening test for depression and anxiety using the complete version of the Patient Health Questionnaire (PHQ-9) and Generalized Anxiety Disorder (GAD-7) may add too much burden if performed on a weekly basis. Therefore, in the updated Mood module, shorter versions of the 2 instruments (PHQ-2 and GAD-2) are used [44,45]. A follow-up evaluation will be performed using the complete version of PHQ and GAD if the screening shows positive results [44,45].

In all 5 modules, a feedback mechanism was added to inform the user of their adherence status.

The other 7 app modules are new. They were added into the iMHere 2.0 system to provide more diverse self-management services to PwCCDs. These include the following:

1. *Exercise*: This module allows PwCCDs to keep track of the exercise and physical activity they perform each day as well as the duration (in minutes) of each. A library of 49 activities (eg, stretching, hand-cycling, gardening, shopping, strolling, and horseback riding) was provided so that the PwCCDs can easily make selections and record the duration of each activity. PwCCDs are also able to add new activities if they are not available in the activity library.
2. *Nutrition*: This module allows PwCCDs to keep track of their food and drink consumption daily by simply checking the serving amount for each. A slightly modified MyPlate program was used to guide PwCCDs’ daily food and drink consumption [46], including water, fruit, vegetables, grains, protein, dairy, cheese, fast food, snacks, and caffeine.
3. *Education*: This module consists of 12 major sections covering topics relevant to supporting self-management routines, such as information about SB, CP, spinal cord injury (SCI), skin integrity, bowel and bladder, exercise, nutrition, time management, relationship, stress management, and anxiety. This module can deliver health-related information to PwCCDs that is tailored to their conditions. Various types of information delivery approaches are included in the Education module, such as text, picture, audio, and video. Self-assessment in the form of quizzes is provided to allow PwCCDs to evaluate their own knowledge.
4. *Goals*: This module allows PwCCDs to keep track of their progress toward their goals and to rate their progress periodically using a 10-point scale.
5. *Personal health record (PHR)*: This module allows PwCCDs to securely manage their own health information, such as medical history, surgical history, past and current medications, allergies, immunization history, family history, and social history. This module was created to encourage PwCCDs to play a more active role in their own health data management and to make frequently used health records readily available when needed.
6. *Supplies*: This module allows PwCCDs to keep track of needed supplies and set up reminders to reorder each supply at an indicated time.
7. *Wheelchair*: This module is a guidebook for wheelchair users. This guidebook contains information about the manual and power wheelchairs, such as information on wheelchair components, guidance on how to set up a wheelchair, and video tutorials about how to master wheelchair use skills.

These app modules are presented here as examples of the diverse self-management services offered by the iMHere 2.0 system. The scalable architecture will allow us to add more app modules easily into this system in the future to meet the needs of other PwCCD populations when needed.

### Caregiver App for Monitoring and Social Support

The iMHere 2.0 caregiver app is a companion app for PwCCD caregivers. The caregiver app mirrors the app modules in the client app so that the caregiver can monitor the status of a PwCCD in each module and then deliver positive reinforcement to the PwCCD in the form of thumbs up symbols and motivational messages. Motivational messages can be selected from prebuilt templates in the caregiver app or custom messages can be entered by the caregiver. In this app, the caregiver can also set up an amount of points to be awarded for meeting each goal to encourage PwCCDs to reach those goals.

There are several different situations that may exist related to the number of caregivers and the type of caregiver. PwCCDs may have no caregiver, 1 caregiver, or multiple caregivers. Caregiving can be provided by family members, friends, or professional (paid) caregivers. In some instances, the paid caregivers are the PwCCDs’ family members or friends. The iMHere 2.0 system was designed to support all of these caregiver situations. Each type of caregiver is implemented as a different role in a role-based access control approach. Once a caregiver becomes a member of the PwCCDs’ care team and chooses 1 specific caregiver role, the corresponding settings are applied to the caregiver app and the caregiver is able to monitor the situation of the PwCCD and provide appropriate positive reinforcement through the caregiver app.

### Customizable User Interface

Significant efforts have been made in the iMHere 2.0 system to make this mHealth system highly flexible, user-friendly, and easy to use by introducing a number of personalization features in the user interface of the client app. For example, PwCCDs can select a desired color theme, avatar, and profile background picture according to their personal preferences. PwCCDs can also choose font size, font style, button size, line and button space, and hand preference according to their needs. All these user interface changes are applied to all the pages in the client app and they are also synchronized to all the devices that the PwCCDs use.

### Evaluation

In total, 2 studies were performed to evaluate the iMHere 2.0 system, one was a usability study and the other was a feasibility study. The protocols of both studies were approved by the Institutional Review Board office at the University of Pittsburgh.

The usability evaluation was performed to identify usability problems. Study participants were recruited in the Greater Pittsburgh area via study flyers posted at various places and an advertisement posted on the Pitt+Me website. The inclusion criteria were age between 18 and 65 years and capable of communicating with investigators in both oral and written English. The usability study was conducted in a controlled laboratory environment. The study participants were asked to perform several tasks on the iMHere 2.0 client app modules. The 3 modules (MyMeds, Skincare, and PHR) used in this study were chosen because they are relevant to most people and include most of the user interface components utilized in the other modules of this app. Therefore, it is reasonable to assume that if study participants were satisfied with the design and implementation in these 3 app modules, they would also be satisfied with the ones having similar design and implementation but different content in the other modules. In this usability study, the iMHere 2.0 client app was first introduced and the 3 modules were briefly demonstrated. Then, the study participants were asked to perform several tasks on the app using the investigator-provided 9.7 inch Apple iPad Air 2 tablet. Their performance was observed, and the comments of study participants were collected. At the end of the session, each of the participants was asked to fill out the Post-Study System Usability Questionnaire (PSSUQ) [47] to report their overall impression of the app. They were subsequently briefly interviewed to allow us to collect further comments and suggestions.

The feasibility evaluation was performed after the usability study to evaluate the extent to which self-management support can be successfully delivered to the intended participants (PwCCDs) via the iMHere app and identify issues that could occur and affect the implementation process [48]. The study participants were recruited from one of the author’s clinics and through referral from our collaborators. The inclusion criteria were PwCCDs with a diagnosis of SB, SCI, CP, or Traumatic Brain Injury and aged 12 years or older. The feasibility study was conducted in the natural environment of the participants using the participants’ personal mobile devices. The participants were first guided on how to install the app on their own devices and how to use the app for self-management. All 12 modules were made available to them. They were encouraged to use the app for about 3 months regularly. Their app usage data were collected and summarized.

## Results

### Dynamic Interactions in the iMHere 2.0 System

**Figure 4:**
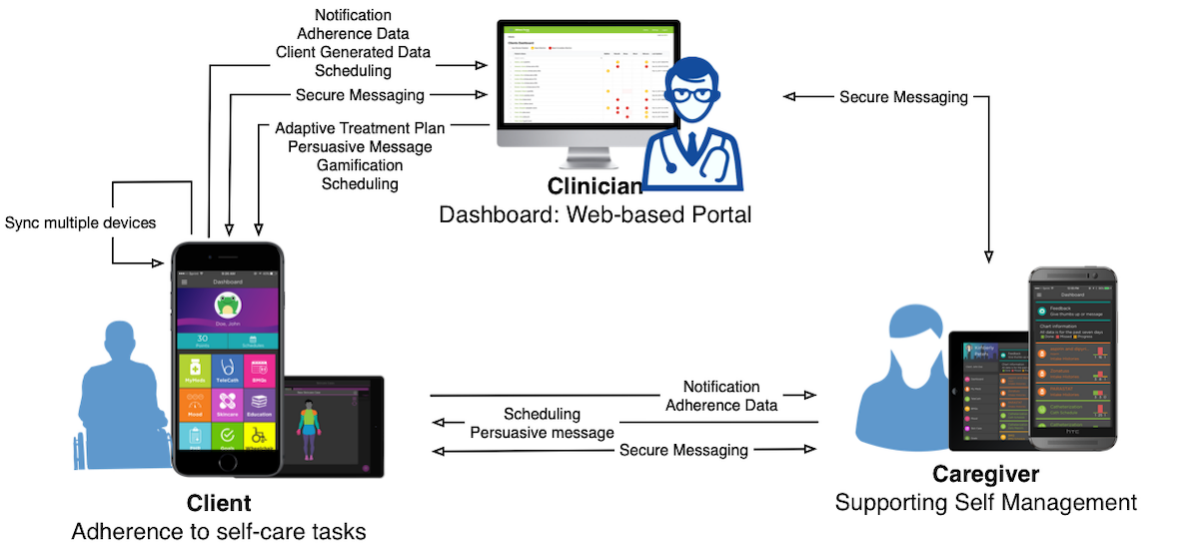
The interactions among the 3 front-end components in the iMHere 2.0 system.

The iMHere 2.0 system consists of 5 components: a cross-platform client app, a cross-platform caregiver app, a Web-based clinician portal, a 2-way secure communication protocol, and a back-end server. The dynamic interactions among the 3 front-end components can be seen in Figure 4. The communication protocol and back-end server transparently support all the activities in these front-end components.

**Figure 5:**
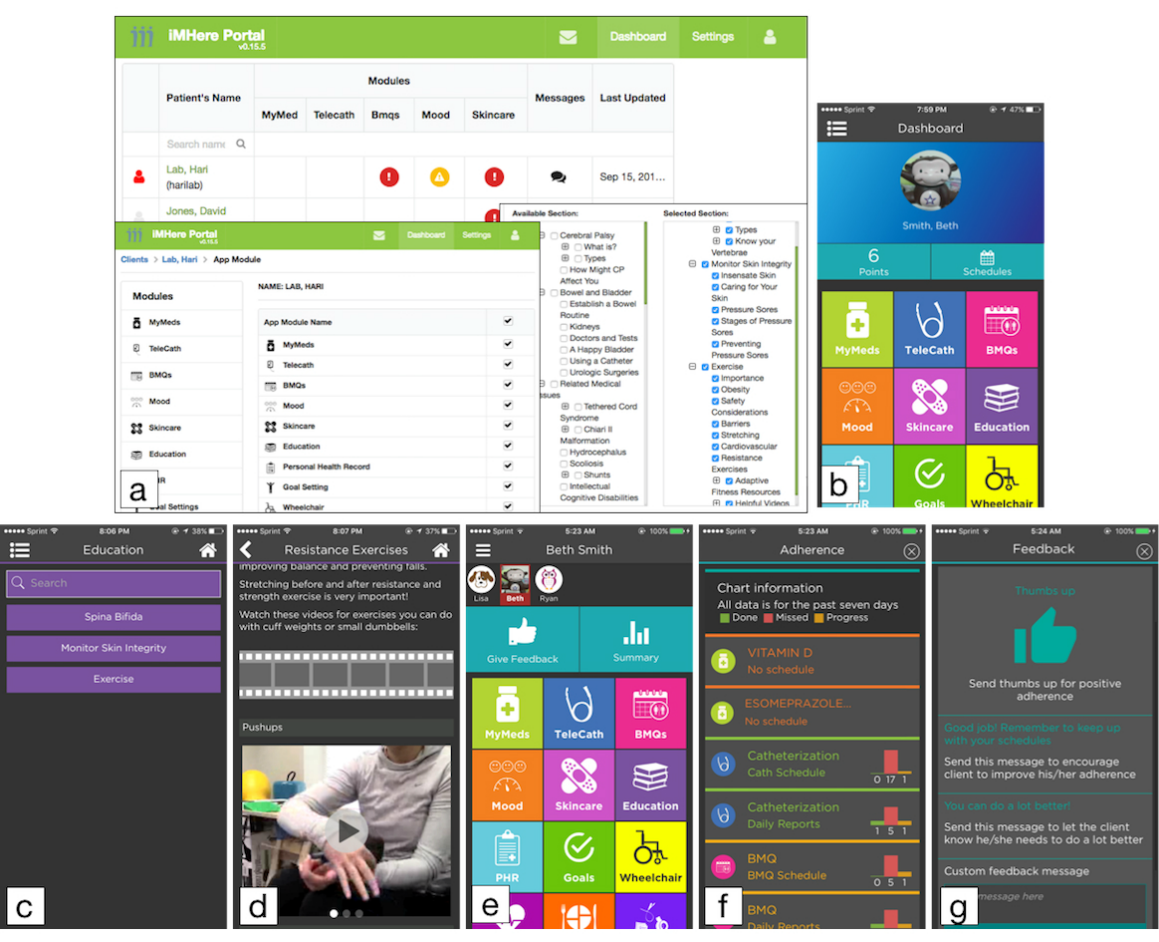
Screenshots of the Web-based clinician portal, the client app, and the caregiver app. (a) The Web-based portal, (b) the dashboard of the client app with chosen app modules, (c) chosen education topics shown in the Education module, (d) an example of a multimedia education page, (e) the dashboard of the caregiver app, (f) the persons with chronic conditions and disabilities’ progress monitoring page in the caregiver app, and (g) the social support page in the caregiver app.

The main components of the Web-based portal are a triage dashboard, patient-context panel, and settings page (Figure 5). The triage dashboard shows a list of PwCCDs with the indicators related to each of the modules selected by clinicians according to PwCCDs’ needs and conditions. These indicators show the severity level and urgency of the PwCCDs’ condition, which can be used by the clinician to determine the priority of the treatment. When an urgent or severe condition occurs while the clinician is not on the Web portal, the iMHere 2.0 system sends notifications to the clinician via short message service text messaging or email so that the clinician can respond to them quickly.

Each PwCCD’s name in the triage dashboard can be used to navigate the clinician to the patient-context panel. The patient-context panel shows all of the components related to the treatment of the PwCCDs, including module management, care team management, and instant messaging.

As a part of the adaptive treatment, in the settings page, clinicians can select the app modules to be used by PwCCDs at any time. In the selected modules, clinicians can provide customized interventions to PwCCDs according to their specific situation. For instance,

- The clinicians are able to select relevant modules based on the baseline evaluation of the client or based on the client’s preferences. Clients are able to request certain modules orally during face-to-face sessions or via the instant messaging service offered in the app.
- When the client reports a problem related to a condition such as a wound (through instant messaging or the Skincare module), the clinicians are able to adapt the existing intervention based on the information provided by the client, such as adjusting the frequency of the skin check reminder.
- If the MyMeds module is selected, clinicians can adjust prescriptions (medication, dosage, and schedule) on the Web-based portal for PwCCDs.
- If the Skincare module is selected, the clinicians can view the pictures of wound sites taken by PwCCDs and provide treatment.
- If the Education module is selected, as patient education materials are arranged into several major sections and subsections, clinicians can choose the relevant education sections and subsections for each of the PwCCDs on the Web-based portal and deploy them as *care bundles* (Figure 5).

All setting changes on the Web-based portal are synchronized with both the client and caregiver apps immediately after they are saved and applied on the portal.

Figure 5 shows how the dashboard of the client app looks corresponding to the app modules selected by the clinician on the Web-based portal. Besides the list of selected modules, this dashboard of the client app also shows patient name, earned reward points, the current day’s schedule, and a chosen avatar on the profile background. Figure 5 shows how the education contents were selected by the clinician in the Web-based portal. Once updated, the education contents were synchronized to the client app. Figure 5 also shows one sample education page, which demonstrates multimedia education content (text, audio, and video).

The layout of the caregiver app is similar to the client app. As a caregiver may have more than one client, the dashboard shows the client roster (Figure 5) and the caregiver can easily switch clients. The dashboard shows a list of modules corresponding to the selected client’s modules. Two buttons are provided to bring up a page with the summary of the client’s progress toward the regimens and a page for positive reinforcement, such as encouragement and thumbs up (Figure 5). The caregiver app allows the caregiver to help the client set up a schedule for self-management tasks, for example, creating or updating schedules for medication taking, mood assessment, and regular skin checking. In addition, the caregiver can set reward points for each of the client’s completed goals to motivate the client toward goal accomplishment.

**Figure 6:**
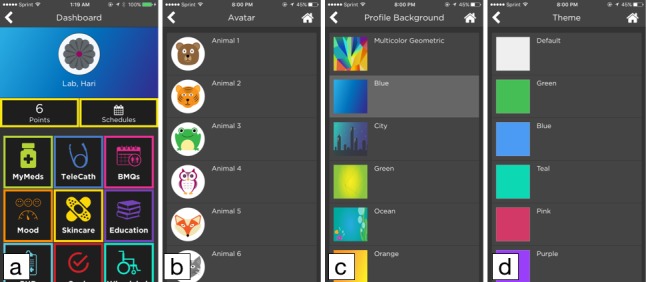
Screenshot of various customization features. (a) The dashboard of the client app with a theme different from the default, (b) a list of avatars, (c) a list of profile background pictures, and (d) a list of themes.

In the client app, each app module is color-coded to help the client identify the correct module while using the app [36]. These sets of colors were arranged into a list of themes for the PwCCDs to choose from. PwCCDs can select 1 theme according to their own preferences. Similarly, there are a list of avatars and a list of profile background pictures. PwCCDs can choose their desired avatar and profile background picture. After these options are selected, the theme, profile background, and avatar are applied to the dashboard of the client app (Figure 6).

### Evaluations

#### Usability Study Results

In total, 81 study participants were recruited in the usability study. Their demographic information is summarized in Table 1.

All 81 participants responded to the 19 statements in the PSSUQ on a 7-point Likert scale, where 1 indicates *Strongly Agree* and 7 indicates *Strongly Disagree*. In other words, a lower number in the answer indicates a stronger agreement with the statement, which corresponds to high usability of the mHealth app in iMHere 2.0. The overall average of responses for PSSUQ was 1.64 out of 7. This means the usability of this mHealth app was high; in other words, these 81 participants believed that the mHealth app was easy to learn and comfortable to use, and they were satisfied with the features provided by this mHealth app.

The feedback from the usability study participants during the informal interview at the end of the usability study was also positive. All 81 participants said they “ *. . .* liked the app,” “it was easy to learn and use the app,” and “the app was very useful for self-management.” Participants specifically liked how easy it was to set up the schedule and reminders and were glad to have the capability to customize the pages and app modules. They also thought that “. . . this app would be useful for all people, not just people with disabilities.”

Some study participants provided suggestions for the further development of the mHealth app in iMHere 2.0, and we made changes in the app accordingly. The following are a few examples:

- In the version used in the study, after a medication schedule was chosen and submitted, there was no message to indicate whether or not the selection was saved. In an updated version, a message was generated to indicate that the medication or schedule had been saved.
- Some icons were used as major app component indicators, but some study participants thought that those icons were clickable and became frustrated after they believed that there was no response from the icons. We chose to remove those icons and added simple texts to label those components so that they would not confuse the client.
- Some participants indicated that it was not intuitive to click the button at the bottom-right corner in MyMeds and Skincare to add medication schedules or report skincare cases because of the large screen of the iPad. A brief instruction was added in the main pages of MyMeds and Skincare to guide the client to use the button at the bottom-right corner.
- Some study participants mentioned other desired features, for instance, tracking of appointments, managing other body symptoms other than skin problems, osteoporosis, and pain, and loading contact information from the contact list to the contacts in the app. We are currently implementing some of these desired features, such as appointment tracking and pain management.

#### Feasibility Study Results

For this feasibility study, 6 participants were recruited. They had all been diagnosed with SB, and their ages were between 23 and 50 years. In total, 2 participants were iPhone users and 4 participants were Android phone users. A total of 90 days’ app usage data for each participant were extracted from the system and analyzed.

The most accessed module during the study was Education (315 times), followed by messaging (116 times). The rest of the modules were accessed 1 to 50 times. The most frequently visited Education sections were *bowel and bladder* (55 times), *monitor skin integrity* (40 times), and *spina bifida* (35 times).

**Figure 7:**
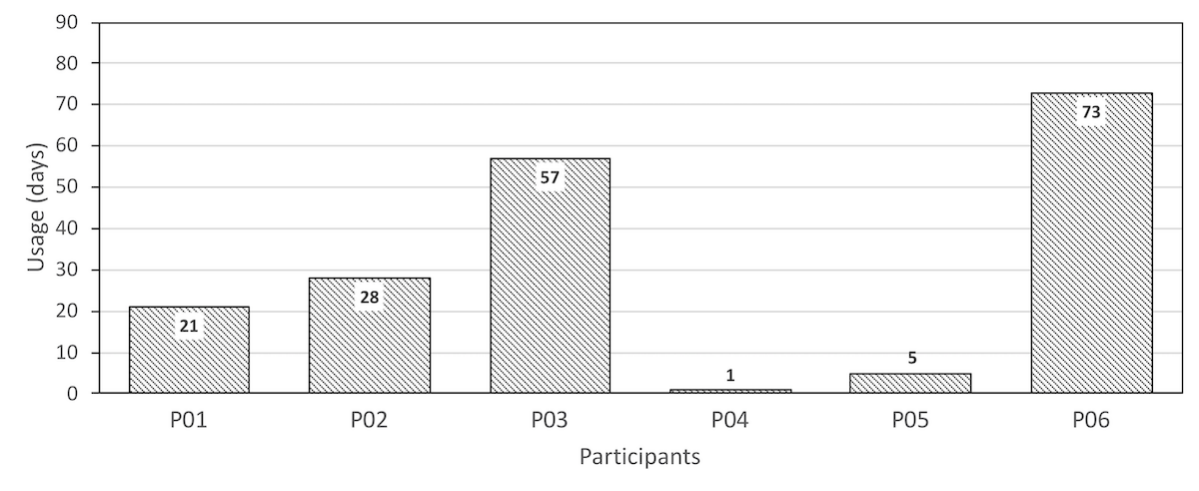
Days the participants actively used the app during the study.

Among these 6 participants, P06 was the most active participant, with 81% (73/90) of the study period actively interacting with the app (Figure 7). P06 was also the participant who was the most compliant to reminders generated by the app, with level of adherence around 88.7% (764/861) based on scheduled reminders during the study period. The frequently visited modules by P01 were Education, messaging, TeleCath, and MyMeds. P04 and P05 lived together and were the least active participants, barely using the app. Unfortunately, it was extremely difficult to reach them using any communication approach (phone, email, text message, or letter) to investigate the reasons behind their underutilization of the app.

P01 and P03 had problems with the reminders. Both participants had part-time jobs and their work schedule was not predictable. This made it difficult for them to set up a regular schedule or respond to the reminders generated according to the schedule. P01 was active in this study for about 23% (21/90) of the time with level of adherence around 38% (5/13) based on scheduled reminders. P03 was active in this study for about 63% (57/90) of the time with level of adherence around 79.1% (349/441) based on scheduled reminders. Both participants frequently visited Education, messaging, and Skincare modules. The different level of interaction in spite of being in the same situation can be explained by the level of support needed by the 2 participants. P01 explicitly mentioned that “...I would not need reminders for certain tasks since I am able to complete the task independently by my own.”

P02 had to stop using the app owing to technical problems. P02 had an old Android device (Samsung S3), which could run an older version of the app but not the newer version, whereas the latter was the one used in the major part of the study. P02 was active for about 31% (28/90) of the study period with level of adherence around 63.3% (112/177) based on scheduled reminders. The most frequently visited modules for P02 were Education, messaging, MyMeds, and TeleCath.

During the study period, participants with Android devices had a number of technical issues, such as incompatible Android versions, interaction problems (screen protector issues, stylus issues, and small keyboard), and the app freezing while in use.

**Table 1:** Demographic characteristics of the study participants (N=81).

Demographic characteristics		Statistics
**Gender, n (%)**		
	Male	34 (42)
	Female	47 (58)
Age (years), mean (SD)		30.4 (12.82)
**Race, n (%)**		
	Black	12 (15)
	White	48 (59)
	Asian	21 (26)
**Education, n (%)**		
	High school	2 (2)
	Some college credits	21 (26)
	Technical training	1 (1)
	Associate degree	4 (5)
	Bachelor’s degree	24 (30)
	Master’s degree	23 (28)
	Professional degree	3 (4)
	Doctoral degree	3 (4)
**Marital status, n (%)**		
	Single	62 (77)
	Married	17 (21)
	Divorced or separated	2 (2)
**Employment, n (%)**		
	Employed	55 (68)
	Not employed	20 (25)
	Retired or disabled	6 (7)
**Household income, n (%)**		
	≤ US $10,000	16 (20)
	US $10,001- US $50,000	33 (41)
	US $50,001- US $100,000	14 (17)
	> US $100,000	11 (14)
	Decline to answer	7 (8)
**Occupation, n (%)**		
	Student	32 (40)
	Researcher	16 (20)
	Administrator	9 (11)
	Customer service	6 (7)
	Other (advisor, attorney, designer, health care provider, teacher, professor, programmer, set up person, unemployed, disabled, or no answer)	18 (22)

With regard to the app, P01 believed that there were too many confirmation steps required when sending a report. More flexible scheduling was preferred by P01, P02, and P03, such as an hourly basis or *every other day* options in MyMeds or an *every two weeks* option in Mood. The app has been modified accordingly by reducing confirmation steps in reporting and adding more flexible scheduling options.

## Discussion

### Principal Findings

To meet the long-term, highly diverse, and changing self-management support needs of PwCCDs, we developed the adaptive mHealth system (iMHere 2.0), which consists of cross-platform client and caregiver apps, a Web-based clinician portal, and a back-end server with a 2-way secure communication protocol. In this system, the adaptive treatment regimens are delivered according to the individual’s specific needs and ongoing performance during treatment [41]. Treatment strategies can be adjusted over time in response to the individual’s performance and needs.

The architecture of the system is highly scalable, which means one can add new self-management services into this system independently if they are needed. In total, 12 highly diverse and commonly used app modules were created in the client and caregiver apps to demonstrate the scalability and flexibility of this system architecture.

The Web-based clinician portal can be used to prescribe personalized treatment strategies for PwCCDs according to their specific conditions and the PwCCDs can follow these personalized instructions in the client app to perform self-management. During the course of the treatment, clinicians are able to adapt the treatment strategies in response to PwCCDs’ performance. Once the treatment strategies are updated by the clinician on the Web-based portal, those strategies are synchronized with the client and caregiver apps. The Web-based portal also enables the clinician to monitor PwCCDs’ adherence to the prescribed treatment strategies and to communicate with the PwCCDs via instant secure messaging.

Social support is also critically important for long-term engagement in self-management [12,49]. In the iMHere 2.0 system, caregivers can conveniently monitor PwCCDs’ performance and provide social support to PwCCDs via the caregiver app. Previous studies have demonstrated that leveraging social influence is an effective strategy to motivate PwCCDs to adhere to treatment regimens [25]. For instance, there is evidence about the significant influence of family members (partners, parents, children, and siblings) on long-term engagement in health care [25]. Therefore, the motivational messages from caregivers may help PwCCDs to endure lengthy treatment procedures. The instant secure messages exchanged between PwCCDs and the clinicians may also provide the desired social support for long-term engagement with the mHealth system.

The availability of multiple caregiver modes makes it feasible for caregivers to provide appropriate support to PwCCDs. For instance, family members may not have formal medical training, but they may have a very close relationship with the PwCCD and extensive knowledge about the PwCCD’s situation. Therefore, the motivational messages from family members mainly show intimacy, love, and encouragement. Paid caregivers typically have some patient care training and, therefore, the motivational messages are mainly professional suggestions and reminders of the potential benefits of consistent self-management. In terms of a caregiver’s access to client’s personal health information, it is desired to have different access levels for different types of caregivers, such as family members and professional caregivers. This feature is currently in our development plan, and it will be implemented in the near future.

Both the client and caregiver apps are cross-platform, which means that these 2 apps can run on the Android, iOS, and Windows Phone systems. In the vast majority of cases, PwCCDs can use the iMHere 2.0 system on their current mobile devices because these 3 mobile operating systems (mainly Android and iOS together) have close to 100% of the global mobile operating system market share [50]. This cross-platform feature also makes it possible for PwCCDs and caregivers to use this system on multiple mobile devices that may have different mobile operating systems or switch mobile operating systems without worrying about losing the opportunity to continue to use the iMHere 2.0 system for self-management.

The data entered by PwCCDs, caregivers, and clinicians are synchronized in real time as long as a network connection is available. If the network connection is not available at some specific moment, PwCCDs can still use most of those app modules as the data are stored locally on the mobile device temporarily and are securely transmitted to a remote secure server using the Secure Sockets Layer protocol once the connection is restored. The only exception is the PHR module, which always requires a network connection as personal medical records are not stored on the local device, even temporarily, to protect the security of patient data. As all the data are stored on a secure remote server, if PwCCDs or caregivers change to new mobile devices, they still can access their complete data.

As the iMHere 2.0 system is for PwCCDs, the accessibility of the app is very important. In iMHere 1.0, some accessibility features were introduced and their use by people with fine motor impairment was studied; the results indicated that participants desired to have the ability to change text size, button size, and color [34]. Therefore, in iMHere 2.0, we introduced these accessibility features so that PwCCDs have the ability to change the font size, font style, button size, space between lines and buttons, and hand preference. These accessibility features are very useful for long-term usage of the iMHere 2.0 system. For instance, when PwCCDs become older, their vision may become worse. If the accessibility features were not available, they might have to switch to a different mHealth app simply because they cannot read the materials in this system anymore. With the accessibility features, they can make adjustments in the settings according to their needs (eg, selecting a larger font size) and so can continue to use the iMHere 2.0 system.

### Comparison With Previous Studies

To our knowledge, this is the first adaptive mHealth platform to provide long-term self-management support for individuals with various types of chronic conditions and disabilities. Intellicare is a suite of 13 mobile apps for depression and anxiety care [51]. These are separate apps and one can choose to install the apps he or she needs. In iMHere 2.0, the client app is one single app and the specific modules can be customized. The biggest advantage of iMHere 2.0 is the instant and adaptive support from caregivers and clinicians, which is not available in Intellicare.

### Limitations

In this paper, we have described the design, development, and evaluation of the iMHere 2.0 system. A usability study was performed with 81 study participants from the general population and the results indicated that the usability of the iMHere 2.0 client app was high. A feasibility study was performed with a small number of PwCCDs and the study participants demonstrated different app module preferences. Some practical issues during implementation were also identified in the feasibility study. A usability study with a group of PwCCDs is currently ongoing. The personalization, especially the accessibility features, has been evaluated in one other study with participants with fine motor impairments. The details about that study will be reported in a separate paper.

Although all of the approaches we implemented in this iMHere 2.0 system have a solid theoretical background and have proved to be effective in certain areas, the effectiveness of this novel approach eventually needs to be determined on a large scale and in long-term randomized clinical trials with participants with various types of chronic conditions and disabilities. One such randomized clinical trial is currently under development, and the results from that trial will be reported in a few years.

In certain circumstances or for some PwCCDs, there is no clinician available to provide care to PwCCDs or to customize app modules. Sometimes, there is no caregiver available, either. Therefore, we created a version of the client app in which the PwCCDs themselves can make selections from available app modules to meet their self-management needs. The evaluation of this version of the client app is currently ongoing.

### Conclusions

The iMHere 2.0 system provides a novel solution in delivering personalized and adaptive preventive interventions to PwCCDs, empowering these individuals to be more independent in managing their conditions with support from clinicians and caregivers. The participants of the usability study were satisfied with the iMHere 2.0 client app and recommended it for a wider target population. The feasibility evaluation revealed different usage and preferences of participants and several practical issues that we need to consider when implementing the system on a larger scale among PwCCDs. This new mHealth system may eventually help PwCCDs prevent many secondary conditions and improve their quality of life.

## Abbreviations

CP: cerebral palsy
GAD: Generalized Anxiety Disorder
iMHere: Interactive Mobile Health and Rehabilitation
mHealth: mobile health
NIDILRR: National Institute on Disability, Independent Living, and Rehabilitation Research
NIH: National Institutes of Health
PHQ: Patient Health Questionnaire
PHR: personal health record
PSSUQ: Post-Study System Usability Questionnaire
PwCCD: persons with chronic conditions and disabilities
SB: spina bifida
SCI: spinal cord injury
